# Can a Village Headman Use an Electronic Village Register and a Simplified Community-Based Verbal Autopsy Tool to Record Numbers and Causes of Death in Rural Malawi?

**DOI:** 10.3389/fpubh.2018.00246

**Published:** 2018-09-04

**Authors:** Chimango V. T. Munthali, Sophie Kang'oma, Khazgani Nasasara, Lindiwe M. Zaina, Chawanangwa Lupafya, Jacob Mziya, Anthony D. Harries, Kudakwashe C. Takarinda, Martha Kwataine, Isaac Dambula, Simeon Yosefe

**Affiliations:** ^1^Baobab Health Trust, Lilongwe, Malawi; ^2^Ministry of Home Affairs and Internal Security, National Registration Bureau, Lilongwe, Malawi; ^3^London School of Hygiene and Tropical Medicine, London, United Kingdom; ^4^AIDS and TB Department, Ministry of Health and Child Care, Harare, Zimbabwe; ^5^Central Monitoring and Evaluation Division, Ministry of Health, Lilongwe, Malawi

**Keywords:** Malawi, deaths, village, electronic village register, verbal autopsy, operational research, SORT IT

## Abstract

**Introduction:** Most people in Africa die without appearing in official vital statistics records. To improve this situation, Malawi has introduced solar-powered electronic village registers (EVR), managed by village headmen, to record birth and death information for production of vital statistics. The EVR is deployed in 83 villages in Traditional Authority Mtema, Lilongwe, which is an area without electricity. In 17 villages, village headmen were also trained to use a simple verbal autopsy (VA) tool adapted from one developed by the World Health Organization (WHO). Study objectives were to (i) document numbers and causes of death occurring in 17 villages between April 2016 and September 2017, and (ii) assess percentage measures of agreement on causes of death as recorded by village headmen using a simple VA tool and by a team of health surveillance assistant (HSA)/medical doctor using the WHO VA tool.

**Methods:** The study was in two-parts: (i) a cross-sectional study using secondary data from the EVR; (ii) primary data collection study comparing causes of death obtained by village headmen using a simple VA tool and by HSA/medical doctor using the WHO VA tool.

**Results:** Over 18 months, 120 deaths were recorded by EVR in 14,264 residents - crude annual death rate 5.6/1,000 population. Median age at death was 43 years with 69 (58%) deaths being in males. Death occurred at home (75%) and at health facility (25%). Malaria, diarrhoeal disease, pulmonary tuberculosis, acute respiratory infection, and stroke accounted for 56% of deaths recorded by village headmen using the simple VA tool. Causes of death between village headmen and the HSA/medical doctor team were compared for 107 deaths. There was full agreement in causes of death in 33 (31%) deaths, mostly for malaria, severe anemia, intentional self-harm, cancer, and epilepsy. Unknown-sudden death and sepsis recorded by the HSA/medical doctor team were responsible for most disagreements.

**Conclusion:** It is feasible for village headmen in rural Malawi to use an EVR and simple VA tool to document numbers and causes of deaths. More work is needed to improve accuracy of causes of death by village headmen.

## Introduction

The systematic recording of death and related causes is a crucial public health component of civil registration and vital statistics, and will be essential for monitoring progress toward targets in the health-related Sustainable Development Goals (SDGs) (1). Indeed, there are two SDG targets and indicators that specifically refer to improving civil registration and vital statistics: by 2030, legal identity will be provided for all people and the proportion of countries that have achieved 100% birth registration and 80% death registration will be documented. New momentum for civil registration and vital statistics is building, driven by increasing demand for accountability and results in health, equity, and rights-based approaches to development (2).

Despite this gathering momentum and the importance of vital statistics, most people in sub-Saharan Africa still die without leaving any trace in legal records or official statistics, and the situation has remained largely unchanged for the last few decades (3, 4). While the recently published systematic analysis of the 2015 global burden of disease study has been an important milestone to understand global, regional and national reasons for death in the last 35 years, there are large uncertainties around some of the estimates in sub-Saharan Africa due mainly to weak or absent registration systems (5). The challenges of unreported deaths are compounded by the fact that the majority of deaths in Africa occur in the community rather than in health facilities (6).

Although these are difficult challenges, there are many benefits to improving civil registration and vital statistics (7). First, the registration of vital events provides individuals with documented evidence of identity, civil status and family relationships. Second, the collected information enables the continuous updating of population registers and identification systems that are central to national administration and security. Third, the registration of births and deaths generates essential population data that underpin policy and planning across many sectors. Finally, there is also evidence to suggest that good performing civil registration and vital statistics systems are associated with better health outcomes because of better evidence-based resource allocation decisions that can enhance individual and collective wellbeing (8).

## Background and rationale

Malawi, a small country in Central-Southern Africa has an estimated population of 17 million and a nominal gross domestic product per capita of USD$250 (9, 10). The country is divided into five health zones and 28 districts. Every district has a number of traditional authorities (TAs), which in turn are composed of villages. Within this structure, there are village headmen who report to group village headmen who in turn report to the TA chief. Each TA reports to the appropriate district commissioner who is in charge of local government.

In 2007, the Malawi Government through the National Registration Bureau (NRB) introduced a registration system for births and deaths based on paper-based village registers, and this reached full country-wide coverage by 2011. These registers were a potential source of vital statistics data at the grass-roots level, but the manual collection, collation and analysis of data from villages up to traditional authorities (TA) and then up to the district commissioner were almost impossible due to poor infrastructure, lack of intermediary human resources and inadequate transport (11).

A potential solution was to develop an electronic village register (EVR) to transmit data through wireless networks from village headmen to the TA Chief to the District Commissioner (DC) and then to the NRB, with this data also shared with the appropriate health facilities. To set up a functioning EVR required addressing several important challenges that included the absence of electricity in rural communities, low literacy levels among village headmen, network infrastructure, and lack of computer skills.

The Baobab Health Trust, a Malawian non-governmental organization involved in the development and implementation of medical and civil registration informatics interventions, took up the challenge and developed a solar-powered EVR in 2013 for two villages in Traditional Authority Mtema (Chalasa and Mtema) in rural Lilongwe, Malawi, where there is no established electricity supply. The pilot study in these two villages showed the feasibility of training a village headman and assistant to use the EVR to document, transmit and collate village demographics along with new births and deaths (12). Following the pilot, the EVR was expanded to all 83 villages in the TA to determine whether an electronic birth and death registration system at village level could be functional and interconnected in this area. The challenges and achievements in making the system robust, user-friendly, and valuable to the community leaders as well as providing information about demographics of village residents and recorded numbers of births and deaths have recently been described (13).

One important missing element, however, has been information on causes of death. In the community, this can only be collected through a verbal autopsy (14, 15). These verbal autopsy questionnaire forms have tended to be long, comprehensive and complicated and have therefore mainly been used in research studies and surveys. In 2012, the World Health Organization (WHO) developed a more user-friendly verbal autopsy tool designed for all age groups, including maternal and perinatal deaths, and this tool uses a simplified cause of death list corresponding to International Classification of Diseases (ICD-10) codes (16). The tool is designed for use by anyone who has completed at least secondary school education, has received good training in conducting a verbal autopsy and who is preferably a community-based health worker.

Community-based health workers are currently overburdened with a multitude of tasks, and given the fact that village headmen have been trained in and use the EVR system, they may be a suitable resource for conducting verbal autopsies and ascertaining causes of death. However, the 2012 WHO verbal autopsy instrument is too complicated and not designed for use by a village headman. A much simpler verbal autopsy tool was therefore designed that adheres to the basic structure and ICD-10 codes of the 2012 WHO verbal autopsy instrument focussing on the most common known causes of death in Malawi.

The aim of this study therefore was to describe how an EVR and a simple community-based verbal autopsy tool could be used by village headmen to record numbers and causes of death in rural Malawi. Among the residents living in TA Mtema between April 2016 and September 2017, the specific objectives were to (i) document the number of deaths during the study period, stratified by age, sex, and place of death as recorded by the village headman in the EVR, (ii) list the causes of these deaths as recorded by the village headman using a simple community-based verbal autopsy and (iii) assess measures of agreement between causes of death documented by the village headmen compared with those obtained from a medical doctor when a community health surveillance assistant (HSA) used the 2012 WHO verbal autopsy tool.

## Essential elements of the intervention

Lilongwe district houses the capital city and is divided into 18 TAs with 221 group village headmen and 2,234 villages, each with a village headman. TA Mtema is the traditional authority site, situated in Lilongwe district, where the study was conducted. This TA is a rural setting with an estimated population of 45,000, with 9 group village headmen and 83 villages. The TA is poor and without electricity. The deployment and scale up of the EVR to an entire traditional authority, including obtaining information on causes of death, was agreed upon and authorized by the NRB of Malawi in 2013. By April 2017, the EVR had been deployed to all 83 villages, connected to nodal servers based at the TA headquarters, the area health center. As with most newly introduced electronic systems in developing countries, the system still relies heavily on routine supervision visits to ensure all vital events are captured. However, we expect this dependency to diminish over time.

### The electronic village register (EVR)

This has been previously described (12), but there has been upgrading and modification of the system which briefly works as follows (13): Power is supplied through solar panels placed close to the village headman's house and these connect to deep-cycle batteries that are enclosed behind a lockable panel that also houses and protects the electronics. All the electronic devices for the EVR are installed on a single modular equipment panel to reduce installation and maintenance efforts in the field, and the system is designed to run 24 h a day. The workstation is custom-constructed out of durable, water-resistant PVC and there is an adjustable mounting bracket that allows easy viewing of the touchscreen computer while standing or sitting. Connectivity between EVRs and nodal servers is achieved using omnidirectional antennas within the wireless mesh network. The whole system is designed to run 24 h a day, with daily support provided through onsite and remote supervision.

The village headman and a designated literate person from the village are in charge of the EVR, with training undertaken by the team from Baobab Health Trust. Once an EVR has been set up, a demographic situational analysis is carried out documenting the number of village residents, stratified by age and sex. This is done by going from door-to-door and either registering villagers in the EVR if they already have a health passport or by issuing a health passport to those who do not have one and at the same time giving them a national identity card. Every time there is a birth and death in the village, this information is entered to the EVR and monthly reports on births and deaths are sent to the nodal servers and collated for a monthly TA report.

### Simple community-based verbal autopsy tool and its deployment in TA mtema

A simple verbal autopsy tool was developed (see Table 1) and adapted from the 2012 verbal autopsy tool designed by the WHO (16). This tool was developed by the team at the Baobab Health Trust working in collaboration with staff at the NRB, and was based on what a village headman might find feasible for use in the field. It was shared and agreed with village headmen before the study commenced. Once the tool had been developed in English, it was translated by the Baobab Health Trust team (all Malawian staff) into the local language Chichewa. Based on the number of deaths recorded in the EVR by September 2017, 17 villages with five or more deaths in the previous 18 months were selected for the study. In each of these villages, a line list with demographic details was made of the deaths that had been recorded between April 1st, 2016 and September 30th, 2017. The village headman and the village secretary (altogether 17 headmen and 17 secretaries) were trained together in the use of this verbal autopsy tool and were trained based on the structure of the tool to decide on the cause(s) of death. After an acceptable time to allow for bereavement and grief before the families were approached, village headmen with or without their assistants visited the families of the deceased, obtained verbal and written consent for participation in the study and then completed the simplified community-based verbal autopsy tool. Following this visit, the families were also informed about and provided consent for a second interview with the HSA.

**Table 1:** Simple verbal autopsy tool for use by village headman.

**Deceased demographic details**				
Unique identifier no from electronic village register. ______________				
Name of village___________________			Verbal autopsy date (dd/mm/yy)___/____/______	
First name of deceased			Date of birth (dd/mm/yy) ___/___/____
Last name of deceased			Date of death (dd/mm/yy) ___/___/____
Gender	Male □	Female □	Place of death	
**Informant details**
First name	Last name	Relationship to deceased	Consent obtained		Consent to be interviewed by the Nurse	
			Yes	No	Yes	No
			Family Member not available		Family Member not available	
Verbal autopsy date (dd/mm/yy): ___/___/____					
Verbal autopsy Interviewer**: ______________________**					
Instructions: Please select the option that best represent the response of the respondent					
**Verbal autopsy code**	**Verbal autopsy title**	**ICD-10 code (to ICD)**	**Interviewee responses**	
	**Infectious and parasitic diseases**			
VAs-01.02	Acute respiratory infection (pneumonia)	J22/J18	Yes	No
Vas-01.03	HIV/ AIDS related death	B24	Yes	No
VAs-01.04	Diarrheal Diseases	A09	Yes	No
VAs-01.05	Malaria	B54	Yes	No
VAs-01.06	Measles	B05	Yes	No
VAs-01.09	Pulmonary Tuberculosis	A16	Yes	No
	**Non communicable diseases**			
VAs-05.02	Diabetes Mellitus	E14	Yes	No
VAs-04.99	Hypertension	I99	Yes	No
VAs-05.02	Asthma	J45 (J46)	Yes	No
VAs-04.02	Stroke	I64	Yes	No
VAs-08.01	Epilepsy	G40	Yes	No
	Cancer	C80-1	Yes	No
	**External causes of death**			
VAs-12.01	Road Traffic Accident	V89	Yes	No
VAs-12.03	Accidental fall	W19	Yes	No
VAs-12.05	Accidental exposure to smoke, fire and flames	X09	Yes	No
VAs-12.06	Contact with venomous animals and plants	X29	Yes	No
VAs-12.08	Intentional self-harm	X84	Yes	No
VAs-12.07	Accidental poisoning /exposures to poisons	X49	Yes	No
VAs-12.09	Assault	Y09	Yes	No
VAs-12.10	Exposure to force of nature	X39	Yes	No
	**Other causes of death**			
VAs-10	Neonatal death	P96	Yes	No
Vas-03.01	Severe anemia	D64	Yes	No
VAs-03.02	Severe malnutrition	E46	Yes	No
VAs-09	Pregnancy related/ within 6 weeks of delivery			
VAs-99	**Unknown causes of death**	R99	Yes	No
	Sudden		Yes	No
	Acute/Sub acute (within 3 months)		Yes	No
	Chronic Illness (> 3 months)		Yes	No
	**Final established cause of death**			

### The WHO verbal autopsy tool and its use in TA mtema

The WHO recommended verbal autopsy questionnaires for the different age groups were translated into the local language Chichewa. These were used by health surveillance assistants (HSA) who carried out interviews a few days after the first interview by the village headman and village secretary. HSAs are an established and certified cadre of health care worker, who receive a formal training within the Ministry of Health for several months, after which they are usually attached to health centers around the country performing public health and outreach services for their communities. The interview by the HSA was conducted with the same informant who participated in the first interview. The HSA also obtained verbal and written consent for this second interview before the verbal autopsy questionnaire was completed. The questionnaires (generally with “Yes” or “No” answers) were completed by the HSAs and then given to the medical doctor based in the Ministry of Health, who was trained in interpreting ICD-10 coding and deciding on causes of death based on the answers obtained with the WHO questionnaire. This doctor then assigned a cause of death and issued a verbal-autopsy cause of death certificate.

## Methods

### Study design

This was a two part study. The first part was a cross-sectional analytic study using already collected secondary data from the EVR to provide information about numbers of deaths and the characteristics of the villagers who died. The second part was a primary data collection study comparing causes of death obtained by village headmen using a simple locally developed verbal autopsy tool and a two-man team of HSA/medical doctor using the 2012 WHO verbal autopsy tool.

### Study population

The study population included all residents of 17 villages in TA Mtema (adults and children) who died between April 1st, 2016 and September 30th, 2017.

### Data variables and data collection

Data variables collected from the EVR and the Verbal Autopsy tools included: number of residents living in each village collected at the time the EVR became functional; EVR recorded deaths; date of death; name of the village where the deceased was resident; age at death; sex; the place of death; the family or other informant who was interviewed; date of death; date of interview by the village headmen and the HSA; and the causes of death according to each verbal autopsy instrument used. The verbal autopsies by both village headmen and HSA/medical doctor were conducted between 24th September and 10th October, 2017.

### Analysis

Data from the paper-based autopsy forms were double-entered into EpiData (version 3.1 for data entry, EpiData Association, Odense, Denmark) and analyzed in STATA 13.1 (Stata Corp, College Station, TX, USA). A descriptive analysis with frequencies, means and proportions was performed on the quantitative data. Measures of agreement on causes of death by village headman and the HAS/medical doctor were assessed for various characteristics. Comparisons were assessed by using the chi square test and Fishers Exact test, and presented as odds ratios (OR) and 95% confidence intervals (CI). Adjusted odds ratios (aOR) were calculated using multivariate logistic regression to adjust for potential confounding effects. Levels of significance were set at 5% (*P* < 0.05).

### Ethics

The deployment and scale up of the EVR in the entire traditional authority to document births, deaths and causes of death was agreed upon and authorized by the NRB of Malawi in 2013. The ethics approval for this study, including the written consent forms, was obtained from the Malawi National Health Science Research Committee.

## Results

### Characteristics of residents who died

There were 120 deaths recorded in the EVR occurring in 14,264 residents during the 18-month study period: crude death rate 5.6 per 1,000 population per year. These deaths occurred in 69 (58%) males and 51 (42%) females, with a median age of 43 years (IQR 19–78 years). The deaths were recorded in 25 (21%) children aged 0–14 years, 53 (44%) adults aged 15–59 years and 42 (35%) elderly persons aged 60 years or above. The place of death was at home in 90 (75%) and in the health facility in 30 (25%).

### Informants, timing of interviews, and causes of death by the village headmen

Village headmen and their secretaries conducted verbal autopsies for all 120 village residents who died. Informants for the causes of death were the spouse in 9 (8%), the parents in 36 (30%), siblings in 30 (25%), other family relatives in 44 (37%), and a non-relative in one. The median time between date of death and date of first interview with the village headmen was 318 days (IQR 170-455; range 7 to 543).

Causes of death by broad category according to the village headmen were: infectious / parasitic illness (*n* = 63, 52%), non-communicable diseases (*n* = 28, 23%), external causes (*n* = 9, 8%), other (*n* = 7, 6%), and unknown (*n* = 13, 11%). More specific causes according to each of these broad categories are shown in Table 2. The five most common recorded causes of death were malaria, diarrhoeal disease, pulmonary tuberculosis, acute respiratory infections and stroke, and these accounted for 56% of all deaths.

**Table 2:** Causes of death documented by village headmen using simple verbal autopsy tool in 17 villages, traditional Authority, Mtema, Malawi: April 2016–September 2017.

**Cause of death**	**n**	**(%)**
**INFECTIOUS AND PARASITIC DISEASES**		
Malaria	20	(16.7)
Diarrheal disease	15	(12.5)
Pulmonary tuberculosis	13	(10.8)
Acute respiratory infection including pneumonia	11	(9.2)
HIV and AIDS related death	4	(3.3)
**NON-COMMUNICABLE DISEASES**		
Stroke	8	(6.7)
Hypertension and other unspecified cardiac diseases	6	(5.0)
Cancer	5	(4.2)
Asthma	5	(4.2)
Epilepsy	3	(2.5)
Diabetes mellitus	1	(< 1)
**EXTERNAL CAUSES OF DEATH**		
Intentional self-harm	3	(2.5)
Exposure to force of nature	3	(2.5)
Accidental fall	2	(1.7)
Assault	1	(< 1)
**OTHER CAUSES OF DEATH**		
Severe anemia	5	(4.2)
Neonatal death	2	(1.7)
**UNKNOWN CAUSES OF DEATH**		
Sudden	7	(5.8)
Chronic illness (>3 months)	5	(4.2)
Acute illness (≤ 3 months)	1	(< 1)
Total	120	

### Timing of interviews and causes of death by HSA/medical doctor

HSAs conducted WHO recommended verbal autopsies on the same household informant as the village headmen for 107 (89%) of all recorded deaths. Informants for the causes of death were the spouse in 8 (8%), the parents in 33 (31%), siblings in 29 (27%), and other family relatives in 37 (34%). The median time between the village headman and HSA interviews was 3 days (IQR, 1–4 days).

Causes of death by broad category according to the HSA / medical doctor were: infectious / parasitic illness (*n* = 54, 50%), non-communicable diseases (*n* = 20, 19%), external causes (*n* = 5, 5%), other (*n* = 5, 5%), and unknown (*n* = 23, 21%). More specific causes according to each of these broad categories are shown in Table 3. The five most common recorded causes of death were unknown-sudden death, malaria, acute respiratory infection, sepsis and cancer, and these accounted for 64% of all deaths.

**Table 3:** Causes of death documented by health surveillance assistants/medical doctor using the WHO verbal autopsy tool in 17 villages, Traditional Authority, Mtema, Malawi: April 2016–September 2017.

**Cause of death**	**n**	**(%)**
**INFECTIOUS AND PARASITIC DISEASES**		
Malaria	17	(15.9)
Acute respiratory infection including pneumonia	11	(10.3)
Sepsis	10	(9.4)
Pulmonary tuberculosis	6	(5.6)
Diarrheal disease	6	(5.6)
HIV and AIDS related death	3	(2.8)
Measles	1	(<1)
**NON-COMMUNICABLE DISEASES**		
Cancer	7	(6.5)
Stroke	5	(4.7)
Chronic obstructive pulmonary	3	(2.8)
Epilepsy	2	(1.9)
Hypertension and other cardiac disorders	1	(<1)
Acute cardiac disease	1	(<1)
Asthma	1	(<1)
**EXTERNAL CAUSES OF DEATH**		
Intentional self-harm	4	(3.7)
Exposure to force of nature	1	(<1)
**OTHER CAUSES OF DEATH**		
Severe anemia	2	(1.9)
Neonatal death	1	(<1)
Severe malnutrition	1	(<1)
Premature birth	1	(<1)
**UNKNOWN CAUSES OF DEATH**		
Sudden	23	(21.5)
Total	107	

### Comparisons between village headmen and HSA

Of the 107 comparisons by the village headmen and HSA, there was full agreement on documented specific causes of death in 33 (31%) cases. The levels of full agreement about specific causes of death in relation to place of death, age group of the deceased, time between death and village headman interview, type of informant and broad categories of death are shown in Table 4. There were higher levels of agreement for deaths occurring in a health facility, for deaths under 15 years of age, when the informant was a spouse or parent and when it was an external cause of death, but none of the differences were statistically significant in either adjusted or unadjusted analysis. The lowest level of agreement was found for unknown causes of death.

**Table 4:** Measures of full agreement on specific causes of death in Traditional Authority Mtema, Malawi, between April 2016 and September 2017 as documented by village headmen and health surveillance assistants/medical doctor.

**Characteristic**	**Total number**	**Agreement between VH and HSA**		**OR**	**aOR**
		**n**	**(%)**	**(95% CI)**	**(95% CI)**
All deaths	107	33	(30.8)		
**PLACE OF DEATH**					
Health facility	27	10	(37)	Reference	Reference
Home	80	23	(28.8)	1.5 (0.6–3.7)	1.4 (0.4–5.0)
**AGE GROUP OF THE DECEASED**					
<15 years	22	10	(45.5)	Reference	Reference
15–59 years	47	13	(27.7)	0.5 (0.2–1.3)	0.7 (0.2–3.1)
≥ 60 years	37	9	(24.3)	0.4 (0.1–1.2)	0.7 (0.1–3.6)
Not recorded	1	1	(100)	–	
**TIME FROM DEATH TO VILLAGE HEADMAN INTERVIEW**					
<6 months	30	8	(26.7)	Reference	Reference
6–12 months	40	14	(35)	1.5 (0.5–4.2)	3.2 (0.9–11.8)
>12 months	37	11	(29.7)	1.2 (0.4–3.4)	1.2 (0.3–4.4)
**HOUSEHOLD MEMBER INTERVIEWED**					
Spouse/parent	40	18	(45.0)	Reference	Reference
Sibling	28	5	(17.9)	**0.3 (0.1–0.8)**	0.2 (0.1–1.0)
Other family relation	39	10	(25.6)	0.4 (0.2–1.1)	0.5 (0.1–1.8)
**BROAD CAUSES OF DEATH**					
Infectious and parasitic diseases	54	18	(33.3)	Reference	Reference
Non-communicable diseases	20	7	(35.0)	1.1 (0.4–3.2)	1.5 (0.4–5.5)
External cause of death	5	4	(80.0)	8.0 (0.8–77)	14.7 (1.0–193)
Other causes	5	2	(40.0)	1.3 (0.2–8.7)	0.8 (0.1–6.4)
Unknown cause of death	23	2	(8.7)	**0.2 (0.0**–**0.9)**	**0.1 (0.0**–**0.6)**

*VH, Village Headman; HSA, health surveillance assistant; OR, odds ratio; aOR, adjusted Odds Ratio; CI, confidence intervals. Significant differences are shown in bold font*.

Comparisons of specific causes of death between village headman and HSA are shown in Table 5. When line-listed according to causes of death by village headman, most agreement occurred for malaria, severe anemia, intentional self-harm, cancer, and epilepsy. The recordings of unknown-sudden death and sepsis by HSA were the ones mainly responsible for the disagreements.

**Table 5:** Comparisons of specific causes of death between Village Headman and HSA: levels of agreement and specified causes of disagreement, Traditional Authority Mtema, Malawi, April 2016–September 2017.

**Cause of death by village headmen**	**N**	**Agreement with HSA**		**HSA causes of death that disagreed with VH**
		**n**	**(%)**	
**INFECTIOUS AND PARASITIC DISEASES**				
Malaria	19	11	(58)	ARI (2); HIV/AIDS (1); PTB (1); Unknown sudden cause of death (2); Sepsis (2)
ARI	10	1	(10)	DD (1); Malaria (2); Asthma (1); Unknown sudden cause of death (3); Sepsis (1); COPD (1)
Pulmonary TB	11	1	(9)	ARI (1); DD (1); Neonatal death (1); Unknown sudden cause of death (3); Sepsis (3); Acute cardiac disease (1)
Diarrheal Diseases	15	4	(27)	ARI (2); HIV/AIDS (1); Malaria (1); Measles (1); Hypertension and other unspecified cardiac conditions (1); Stroke (1); Cancer (2); Severe Malnutrition (1); Sepsis (1)
HIV/AIDS	3	1	(33)	Malaria (1); PTB (1)
**NON-COMMUNICABLE DISEASES**				
Cancer	5	3	(60)	Unknown sudden cause of death (2)
Stroke	7	2	(29)	ARI (2); Malaria (1); PTB (1); Unknown sudden cause of death (1)
Epilepsy	3	2	(67)	Unknown sudden cause of death (1)
Hypertension and other cardiac conditions	5	0	(0)	ARI (1); Stroke (1); Unknown sudden cause of death (1); Sepsis (2)
Asthma	4	0	(0)	Stroke (1); Unknown sudden cause of death (1); COPD (2)
**EXTERNAL CAUSE OF DEATH**				
Intentional self-harm	3	3	(100)	
Exposure to forces of nature	3	1	(33)	Unknown sudden cause of death (2)
Accidental fall	2	0	(0)	Unknown sudden cause of death (2)
Assault	1	0	(0)	Intentional self-harm (1)
**UNKNOWN CAUSE OF DEATH**				
Neonatal Death	1	0	(0)	Prematurity (1)
Unknown sudden cause of death	5	2	(40)	Malaria (1); Cancer (1); Sepsis (1)
Unknown acute cause of death	1	0	(0)	Unknown sudden cause of death (1)
Unknown chronic cause of death	5	0	(0)	ARI (2); Cancer (1); Unknown sudden cause of death (2)
Severe anemia	4	2	(50)	PTB (2)
Total	107	33	(31)	

*HSA, Health Surveillance Assistant; DD, Diarrheal disease; HIV, Human Immunodeficiency Virus; AIDS, Acquired Immunodeficiency Syndrome; COPD, Chronic Obstructive Pulmonary Disease; ARI, Acute Respiratory Infection; PTB, Pulmonary Tuberculosis*.

## Discussion

This is the first study to assess the use of a village headman, an EVR and an appropriately simplified verbal autopsy tool to report on deaths and causes of death in a rural setting in sub-Saharan Africa which is poor and has no electricity. There were some encouraging findings.

Using the EVR, 120 deaths were documented in 17 villages within a period of 18 months, with data recorded that included gender, age and place of death. The crude 12-month death rate in the 17 villages was 5.6 per 1,000 per annum, slightly lower than the estimated crude death rate for the country as a whole at 7.3 per 1,000 population in 2016 and 7.1 per 1,000 population in 2017 (17). Whether this means that some of the deaths were not reported/recorded or that the rural population in this setting had a lower crude death rate due for example to less HIV-infection is not clear, and requires further investigation. In rural Malawian communities, deaths have to be reported to the village headman so that a plot for burial is provided, and failure to do this results in having to pay a fine. Thus, it is likely that most deaths were reported but we do not know whether these were all recorded in the EVR by the village headman. The study also found that the majority of deaths occurred at home, which is in line with other information obtained from a district in the Southern Region several years ago (18). The patterns of death, and especially the predominance of death in males compared with females, accords with what was reported in Malawi's most recent Demographic and Health Survey (19).

The village headmen and their secretaries, after receiving training in the simplified verbal autopsy tool, were also able to visit the homes of those who had died, complete the questionnaire and decide on a cause of death. Three quarters of all deaths recorded by village headmen were due to communicable and non-communicable diseases. Malaria, diarrhoeal disease and respiratory infections (pulmonary tuberculosis and acute respiratory infections combined) accounted for most communicable diseases while stroke, cardiovascular disease/hypertension, cancer, and asthma accounted for most non-communicable diseases. When the HSA/medical doctor collected information on causes of death using the WHO autopsy tool they recorded that about 70% of deaths were also due to communicable and non-communicable diseases, with external/other causes accounting for 10% and unknown-sudden death for the remainder. These two broad patterns generally accord with CDC estimates of the top ten causes of death in Malawi (20), except for HIV/AIDS. The few cases of HIV/AIDS documented by both village headmen and HSA were surprising, given the extent of the HIV/AIDS epidemic in Malawi (21), and this may be due to stigma and confidentiality issues preventing this disease being discussed in a rural village setting.

In terms of one on one comparisons based on unique ID, there was agreement between village headman and HSA/medical doctor in only one third of deaths. This low level of agreement may not be too surprising. The two verbal autopsy tools were different with the WHO tool requiring boxes to be ticked off on multiple pages and these then collated to give a final cause of death decided upon by a medical doctor sitting in the Ministry of Health. Moreover, the WHO tool contained causes of death that were not available in the village autopsy tool, such as sepsis, premature death and chronic obstructive pulmonary disease. When the village headman verbal autopsy tool was developed, only common diseases which could be identified and recognized by laypersons were listed, and sepsis was not included. Deciding on whether a death is due to asthma or chronic obstructive pulmonary disease, acute respiratory infection or pulmonary tuberculosis, HIV-related disease or fever / tuberculosis can be difficult even in the best of circumstances. A systematic review and meta-analysis of post-mortem studies of HIV-infected adults in resource-limited settings showed that tuberculosis (largely disseminated in nature) was the cause of death in 37% of adult AIDS-related deaths, with the disease being unrecognized or undiagnosed at death in almost half of the cases (22). Finally, about 20% of deaths recorded by HSA/medical doctor were attributed to unknown-sudden death. How this cause of death was decided upon is not clear and requires further in-depth understanding as we move forward with this community-based initiative.

The strengths of this study were that TA Mtema and its villages are typical of the many TAs and 25,000 villages scattered throughout Malawi. The potential therefore for replicating this initiative in other parts of the country is high. Both verbal autopsy forms used standardized ways of documenting final causes of death, based on ICD-10 coding, and the same informant was always interviewed by village headman and HSA. The study was also reported according to STROBE guidelines (Strengthening the Reporting of Observational Studies in Epidemiology) (23).

There were some important limitations. Matched interviews were conducted for 107 rather than 120 deaths, the main reason being that the HSA could not find the same informant when visiting the village. Because the EVR was functional up to 18 months before the verbal autopsies were carried out, there was a long time span from date of death to the first interview which may have led to recall bias of the true cause of death. There was also no second doctor to validate the cause of death diagnosed using the WHO verbal autopsy tool.

There have been many previous studies reporting on the standardization of verbal autopsy tools. These tend to use different methodologies and utilize two or more trained physicians to review the forms and determine a cause of death which is then coded using the WHO ICD-10 classification of disease. The physician identified codes are then compared with hospital diagnoses or death certificates as the gold standard (24–29). These defined categories of causes of death coded by each physician and the percent agreement are usually compared using the kappa statistic (30). We felt that this methodology was not appropriate in our study because of different variables being present in the two verbal autopsy tools, and therefore we compared measures of agreement and assessed whether these were affected by certain characteristics such as type of informant, place of death and age of deceased using simple chi square tests and Fisher's exact test for statistical significance.

### Lessons learned

There are two important lessons to be learned from this study. First, we have shown that village headmen and their secretaries can use an EVR to numerically document deaths in rural villages and that these EVR can function in a poor area with no electricity. An update on the status of the EVR in all 83 villages in TA Mtema describes how modifications have been made to the system over time and the value propositions of the EVR for the village headmen which include daily postings of news and sports items and sockets for charging mobile phones and lanterns (13). Despite a high total equipment cost of about USD$2,500 per village (13), this information suggests that village leaders and the community can participate in the collection and transmission of vital statistics, provided value propositions are discussed, agreed and acted upon, and this needs to be considered by policy makers involved in getting civil registration and vital statistics systems off the ground.

Second, we have also shown that village headmen and their secretaries can use a simplified verbal autopsy tool to record causes of death that align with ICD-10 codes. What we are unsure about is the accuracy of these causes of death. Our comparative design using the HSA / medical doctor and the 2012 WHO verbal autopsy tool was problematic with agreement on specific causes of death being just over 30%. This is too low to decide on further expansion to other villages, and we need to discuss, review and consider further modification of the village headman autopsy tool along with modified and refresher training. This will require some prospective qualitative research focused on village headmen and informants of persons who died to understand what they felt about the tool and its ease of use and the appropriateness of the training which they received. We also need to understand more about the importance that the Ministry of Health attaches to documenting causes of death and the decision making process of the HSA/medical doctor and in the future we need to consider setting up quarterly face-to-face meetings to discuss the differences in causes of death obtained by village headman and HSA. Moving forward, the long time periods between death and village headmen interview need to be avoided.

## Conclusion

This study in 17 rural villages in TA Mtema, Lilongwe, Malawi, showed that village headmen were able to use an EVR to numerically document the numbers of deaths, and the EVR system has already been expanded to the 83 villages in TA Mtema. The EVR is expensive as a stand-alone system, so expansion to other traditional authorities in the country or elsewhere will probably be dependent on additional functionalities such as health care support and social development. The study has also shown that village headmen can use a simple verbal autopsy tool to document causes of death. However, there was full agreement in only about one third of deaths when village headmen causes of death were compared with those recorded from the same informants by HSAs/medical doctor. The use of village headmen to obtain causes of death is not ready therefore for further expansion within or beyond the traditional authority as there is need to understand the reasons for the differences in causes of death and provide solutions for better agreement. Nevertheless, overall these are encouraging findings that suggest that village leaders and the community can usefully participate in civil registration and vital statistics systems.

## Data availability statement

Data sets are available on request. The raw data that support the results and conclusions of the study can be made available, without undue reservation, to any qualified researcher.

## Author contributions

CM and AH conceived the study. CM, AH, and KT designed the study protocol. CM, SK, KN, LZ, CL, JM, AH, KT, MK, ID, and SY read and approved the protocol; contributed to analyzing and interpreting the data; critically revised the manuscript for intellectual content; read and approved the final manuscript. CM, KN, LZ, JM, and SY collected the data. CM, AH, and KT drafted the manuscript.

### Conflict of interest statement

The authors declare that the research was conducted in the absence of any commercial or financial relationships that could be construed as a potential conflict of interest.
